# Supplementary material for: Reference Gene Validation for RT–qPCR in PBMCs from Asthmatic Patients with or without Obesity
Source: Methods Protoc. 2022 Apr 22;5(3):35. doi: 10.3390/mps5030035 (PMC9149987; doi:10.3390/mps5030035)
Supplement: Supplementary file 1 [file mps-05-00035-s001.zip › mps-1648780-supplementary.pdf]

methods and protocols

MDPI

Supplementary Material

1 Supplementary Tables

Table S1. Patients' clinical features.

	Healthy	Asthmatic	Obese asthmatic	p value
N,	3	3	3	-
**Women**, n	2	2	3	ns
**Age**, years	50 ± 14	53 ± 16	51 ± 2	ns
**BMI**, kg/m^2	25.5 ± 3	24.9 ± 3	47 ± 5	< 0.001
**FEV_1**, %	99.3 ± 22	88.3 ± 23	88.7 ± 9	ns
**FEV_1 (post)**, %	103 ± 23	94 ± 17	93.3 ± 3	ns
**FEV_1 / FVC**, %	73 ± 11	65.3 ± 8	82.7 ± 1	ns
**FEV_1 / FVC (post)**, %	75 ± 9	71.7 ± 4	85.7 ± 1	ns
**FeNO**, ppb	35.7 ± 20	54.7 ± 18	23.3 ± 14	ns
**25-OH VitD**, ng/ml	24 ± 12	24 ± 4	15 ± 8	ns

Data expressed as mean ± SD. BMI: body mass index; FEV_1: forced expiratory volume in 1 second; FVC: forced vital capacity; FeNO: factional exhaled nitric oxide; 25-OH VitD: 25-hydroxyvitamin D. One-way ANOVA with post-hoc Tukey's multiple comparison tests were used.

Table S2. Reference genes evaluated in this study.

Symbol	TaqMan Assay ID	Protein name	Accession number	Amplicon length (bp)
GUSB	Hs99999908_m1	Glucuronidase beta	NM_000181	81
B2M	Hs00187842_m1	Beta-2-microglobulin	NM_004048	64
POLR2A	Hs00172187_m1	RNA polymerase II subunit A	NM_000937	61
PPIA	Hs99999904_m1	Peptidylprolyl isomerase A	NM_001300981	98
ACTB	Hs01060665_g1	Actin beta	NM_001101	63
GAPDH	Hs02758991_g1	Glyceraldehyde-3-phosphate dehydrogenase	NM_001256799	93
HPRT1	Hs02800695_m1	Hypoxanthine phosphoribosyltransferase 1	NM_000194	82
TBP	Hs99999910_m1	TATA-box binding protein	NM_003194	127

Table S3. Quantification cycle values in healthy, asthmatic, and obese asthmatic PBMCs.

	**Healthy**	**Asthmatic**	**Obese asthmatic**	Kruskal-Wallis	Dunn's multiple comparisons test		
				p value	**H vs A**	**H vs OA**	**A vs OA**
GUSB	26.73 ± 0.58	27.88 ± 0.91	25.94 ± 0.99	< 0.0001	0.0009	0.0047	<0.0001
B2M	18.75 ± 0.67	20.51 ± 0.76	18.86 ± 0.80	< 0.0001	<0.0001	>0.9999	<0.0001
POLR2A	26.26 ± 0.61	27.68 ± 0.79	25.42 ± 1.04	< 0.0001	0.0010	0.0005	<0.0001
PPIA	20.59 ± 0.82	23.44 ± 0.76	20.50 ± 0.96	< 0.0001	<0.0001	>0.9999	<0.0001
ACTB	21.11 ± 0.67	22.81 ± 1.03	20.80 ± 1.15	< 0.0001	<0.0001	0.6067	<0.0001
GAPDH	22.55 ± 1.07	24.48 ± 1.02	21.73 ± 1.27	< 0.0001	0.0005	0.1015	<0.0001
HPRT1	28.75 ± 0.64	30.33 ± 1.00	28.07 ± 1.03	< 0.0001	0.0003	0.1538	<0.0001
TBP	28.26 ± 0.91	29.64 ± 0.90	27.68 ± 0.91	< 0.0001	0.0008	0.0390	<0.0001

Data expressed as median ± SD. H: healthy subjects; A: asthmatic patients; OA: obese patients. Kruskal–Wallis with post hoc Dunn’s multiple comparison tests were used.
